# Prognostic value of pre-therapeutic nutritional risk factors in elderly patients with locally advanced esophageal squamous cell carcinoma receiving definitive chemoradiotherapy or radiotherapy

**DOI:** 10.1186/s12885-023-11044-5

**Published:** 2023-06-28

**Authors:** Jianjian Qiu, Jun Yang, Yilin Yu, Zhiping Wang, Hancui Lin, Dongmei Ke, Hongying Zheng, Jiancheng Li, Qiwei Yao

**Affiliations:** Clinical Oncology School of Fujian Medical University, Fujian Cancer Hospital, Fuzhou, 350014 China

**Keywords:** Elderly esophageal squamous cell carcinoma, Pre- therapeutic nutrition-related indicators, Risk stratification, Survival

## Abstract

**Background:**

The nutritional status of cancer patients is a crucial factor in determining their prognosis. The objective of this study was to investigate and compare the prognostic value of pretreatment nutrition-related indicators in elderly esophageal squamous cell carcinoma (ESCC). Risk stratification was performed according to independent risk factors and a new nutritional prognostic index was constructed.

**Methods:**

We retrospectively reviewed 460 older locally advanced ESCC patients receiving definitive chemoradiotherapy (dCRT) or radiotherapy (dRT). This study included five pre- therapeutic nutrition-related indicators. The optimal cut-off values for these indices were calculated from the Receiver Operating Curve (ROC). Univariate and multivariate COX analyses were employed to determine the association between each indicator and clinical outcomes. The predictive ability of each independently nutrition-related prognostic indicator was assessed using the time-dependent ROC (time-ROC) and C-index.

**Results:**

Multivariate analyses indicated that the geriatric nutrition risk index (GNRI), body mass index (BMI), the controlling nutritional status (CONUT) score, and platelet-albumin ratio (PAR) could independently predict overall survival (OS) and progression-free survival (PFS) in elderly patients with ESCC (all *p* < 0.05), except for prognostic nutritional index (PNI). Based on four independently nutrition-related prognostic indicators, we developed pre-therapeutic nutritional prognostic score (PTNPS) and new nutritional prognostic index (NNPI). No-risk (PTNPS = 0–1 point), moderate-risk (PTNPS = 2 points), and high-risk (PTNPS = 3–4 points) groups had 5-year OS rates of 42.3%, 22.9%, and 8.8%, respectively (*p* < 0.001), and 5-year PFS rates of 44.4%, 26.5%, and 11.3%, respectively (*p* < 0.001). The Kaplan–Meier curves showed that the mortality of elderly ESCC patients in the high-risk group was higher than that in the low-risk group according to the NNPI.

Analysis of time-AUC and C-index revealed that the NNPI (C-index: 0.663) had the greatest predictive power on the prognosis in older ESCC patients.

**Conclusions:**

In elderly ESCC patients, the GNRI, BMI, CONUT score, and PAR can be used as objective assessment measures for the risk of nutrition-related death. Compared to the other four indexes, the NNPI has the greatest prognostic value for prognosis, and elderly patients with a higher nutritional risk have a poor prognosis, which is helpful in guiding early clinical nutrition intervention.

**Supplementary Information:**

The online version contains supplementary material available at 10.1186/s12885-023-11044-5.

## Introduction

Esophageal cancer (EC) is the sixth leading cause of cancer-related death globally, with esophageal squamous cell carcinoma (ESCC) being the most common pathological type in Asia [1]. Despite significant advances in effective new treatments (such as immunotherapy) and diagnostic strategies, the overall 5-year survival rate for EC is only 15–25% [2]. It is well known that EC mainly occurs in middle-aged and elderly patients, with 30% of patients being over 75 years old at diagnosis [3, 4]. With the improvement of living and medical standards, there are more and more elderly EC patients. Furthermore, elderly patients with EC are more likely to be malnourished at the time of diagnosis, and malnutrition is closely related to a poor prognosis [5]. However, in clinical practice, its importance is often underestimated due to a lack of awareness and the lack of effective nutritional risk screening tools [6].

Numerous studies have proven that the progression and metastasis of cancer depend not only on the pathological type of the tumor, therapeutic approaches, tumor staging, etc. [7], but also on the nutritional status of the patients [8–10]. Multiple nutrition-related indicators, such as the geriatric nutrition risk index (GNRI), body mass index (BMI), the controlling nutritional status (CONUT) score, the platelet-albumin ratio (PAR), and the prognostic nutritional index (PNI), have recently been shown to be valid predictors of prognosis in EC. A meta-analysis of many human cancers found that low GNRI was related with poor overall and cancer-specific survival [11]. In the past, serum albumin and BMI were widely used to evaluate the nutritional condition of cancer patients. The CONUT score is a new prognostic indicator for numerous cancer types, including ESCC [12, 13]. Recent studies indicate that a low PNI and a high PAR, which are composed of serum albumin and lymphocytes or platelets, are useful prognostic parameters for ESCC [14–16]. Importantly, the nutrition-related parameters listed above can be thoroughly estimated using clinically objective data, such as height, weight, albumin, cholesterol, lymphocytes, etc. This objective and straightforward method for monitoring the nutritional condition of elderly people should be extensively adopted in clinical settings.

The elderly are one of the most susceptible and heterogeneous patient populations, with a greater risk of malnutrition [17]. Furthermore, progressive dysphagia is the primary clinical symptom of EC in the majority of patients [18]. Therefore, it is necessary to assess older EC patients with a greater nutritional risk in order to provide appropriate therapies that decrease malnutrition-related mortality and lengthen survival. This study sought to determine the association between pre-treatment nutrition-related indicators and the prognosis of older patients with ESCC who received definitive chemoradiotherapy (dCRT) or radiotherapy (dRT). In addition, we developed a pre-therapeutic nutritional prognostic score (PTNPS) and novel nutritional prognostic index (NNPI) based on independent nutritional risk variables. Lastly, the prognostic prediction ability of nutritional prognostic indicators was compared, and the nutritional risk of the patient population was stratified based on PTNPS.

## Material and methods

### Participants

Between March 2011 and August 2020, 460 patients with locally advanced ESCC who received dRT or dCRT at the Fujian Provincial Cancer Hospital were included. In this retrospective study, the criteria for inclusion were as follows: (i) age ≥ 65 years; (ii) histopathology confirmed ESCC; (iii)absence of surgery; (iv) no distant metastasis or 2 or more primary tumors; and (v) availability of relevant laboratory test data before treatment (including routine biochemical tests and routine blood tests). The exclusion criteria were as follows: (i) patients with serious diseases, such as severe infections and immunodeficiency syndrome; (ii) no completed radiotherapy or radiotherapy that has been interrupted for more than 7 days; (iii) no complete follow-up information; and (iv) no signed informed consent. Finally, 460 older patients with locally advanced ESCC (stages II-IVA) matched the aforementioned inclusion criteria. All patients used the 8^th^ edition of the tumor, node, and metastasis (TNM) classification for clinical staging. This single-institutional study conformed to the Declaration of Helsinki and was sanctioned by Fujian Cancer Hospital 's Ethics Committee.

### Treatment and follow-up

All patients underwent a radiotherapy-based treatment plan. Based on their individual condition, patients underwent dRT or dCRT to a total of 50–70 Gy by either intensity-modulated radiotherapy (IMRT) or 3-dimensional conventional radiotherapy (3D-CRT). All treatment plans were carried out using the Philips Pinnacle system, and the radiation techniques were delivered with 6-MV X-ray accelerators with a daily fraction of 1.8–2.0 Gy and five fractions each week. Target volumes and Organs at risk (OAR) were defined in accordance with the 2019 guidelines of the National Comprehensive Cancer Network. 65% of patients were treated with chemotherapy. The common protocol for chemotherapy was as follows: 5-FU (D1-2) / cisplatin (D2) regimen, docetaxel (D1) or paclitaxel (D1) + cisplatin (D2), nedaplatin (D2), carboplatin (D2), or lobaplatin (D2) regimen.

To monitor therapy-related toxicities and the patient’s general condition, all patients were checked weekly during treatment. Following completion of dRT or dCRT, patients were followed up every three months for the first two years and then every six months thereafter. Physical examination, chest computed tomography (CT), barium swallow, 18F-fluorodeoxyglucose positron emission tomography (FDG-PET), and tumor indicators were included in the follow-up evaluation.

### Data collection and calculation of pre-treatment nutritional status

The baseline characteristics of patients included sex, age, tumor location, tumor length, T stage, N stage, and TNM stage. In addition, the patient’s weight and height were measured before the initial treatment. One week before to therapy, routine blood biochemical data was gathered, including total cholesterol and albumin levels, platelet and lymphocyte counts. We obtained nutrition status related indicators based on the above variables, namely, the controlling nutritional status (CONUT), body mass index (BMI), platelet-albumin ratio (PAR), prognostic nutritional index (PNI), and geriatric nutrition risk index (GNRI). These nutritional indicators were calculated as follows: the CONUT score = albumin level [≥ 35 g/dL (0 point), 30–34 g/dL (2 points), 25–29 g/dL (4 points), or < 25 g/dL (6 points)] + the lymphocyte counts [≥ 1600 count/mm^3^ (0 point), 1200–1599 count/mm^3^ (1 point), 800–1199 count/mm^3^ (2 points), < 800 count/mm^3^ (3 points)] + total cholesterol level [≥ 180 mg/dL (0 point), 140 -179 mg/dL (1 point), 100–139 mg/dL (2 points), < 100 mg/dL (3 points)]; BMI = weight (kg)/height (m)^2^; PAR = absolute platelet count/albumin level; PNI = 5*lymphocyte count + albumin level; GNRI = 1.489*albumin (g/L) + 41.7*[(current weight (CW)/ideal body weight (IBW)). The IBW was determined using the formula height (m^2^) * 22 (kg/m^2^). When the CW exceeded the IBW, the ratio of CW/IBW was set to 1.

### Endpoints

The study’s primary endpoint was overall survival (OS), calculated as the time between the pathological diagnosis and death. The secondary endpoint was progression-free survival (PFS), defined as the time from pathological diagnosis to tumor progression and death. For patients who died before the conclusion of the study, the date of death was considered the study endpoint; for those who survived, the date of the final follow-up was considered the study endpoint. The present study’s final follow-up date is February 2022.

### Statistical analysis

Statistical analyses were conducted using IBM SPSS software 25.0 and R software 4.0.2. We turned all continuous variables into classified variables according to the optimal cut-off value of the Receiver Operating Curve (ROC). Using the Chi-square test or Fish’s exact test, the difference between the classified data was evaluated. OS and PFS were plotted using the Kaplan–Meier curves and compared using the log-rank test. The univariate and multivariate Cox analyses were performed to evaluate the association between pre-treatment nutrition-related indicators and prognosis in older patients with locally advanced ESCC. In univariate analyses, all variables with a *p* value < 0.10 were incorporated into multivariate analyses. Using the time-dependent ROC (time-ROC) and C-index, the predictive ability of each independently nutrition-related prognostic indicator was evaluated. In addition, the relationship between nutrition-related indicators and survival outcomes was assessed using the Restricted Cubic Splines (RCS). To understand the application of the same prognostic indicators in different subgroups, we performed a subgroup analysis of age, sex, radiation dose, chemotherapy, tumor location, tumor length, T stage, N stage, tumor stage, and PNI. Statistically, a *p* value < 0.05 was considered significant.

## Results

### Clinical characteristics

In total, 460 elderly patients participated in the study. The baseline characteristics of patients are shown in Table 1. Among them, the majority were males (65.9%), and 58.9% were younger than 76 years old. The majority of the patient’s tumors were located in the middle and lower esophagus regions. The majority of patients underwent chemotherapy (65.0%), and the tumor stage was II for 114 patients (24.8%) and III/IVA for 346 patients (75.2%). Chemotherapy recipients were typically younger and more likely to be in stage IVA. The optimal cut-off values for tumor length, RT dose, BMI, GNRI, CONUT, PAR, and PNI were 5.9 cm, 59.96 Gy, 19.51, 96.36, 3, 5.33, and 46.55, respectively.

**Table 1:** Baseline characteristic in 460 elderly patients with locally advanced esophageal squamous cell carcinoma

Characteristic	No. of Patients
Sex	
Male	303 (65.9%)
Female	157 (34.1%)
Age (years)	
< 76	271 (58.9%)
≥ 76	189 (41.1%)
Tumor location	
Cervical/upper	135 (29.3%)
Middle/lower	325 (70.7%)
Tumor length (cm)	
< 5.9	273 (59.3%)
≥ 5.9	187 (40.7%)
RT dose (Gy)	
< 59.96	118 (25.7%)
≥ 59.96	342 (74.3%)
Chemotherapy	
No	161 (35.0%)
Yes	299 (65.0%)
T stage	
T2/3	273 (59.3%)
T4	187 (40.7%)
N stage	
N0/1	351 (76.3%)
N2/3	109 (23.7%)
Tumor stage	
Stage II	114 (24.8%)
Stage III/IVA	346 (75.2%)
BMI	
< 19.51	142 (30.9%)
≥ 19.51	318 (69.1%)
GNRI	
< 96.36	236 (51.3%)
≥ 96.36	224 (48.7%)
CONUT	
< 3	353 (76.7%)
≥ 3	107 (23.3%)
PAR	
< 5.33	186 (40.4%)
≥ 5.33	274 (59.6%)
PNI	
< 46.55	206 (44.8%)
≥ 46.55	254 (55.2%)

*RT* Radiotherapy, *BMI* Body mass index, *GNRI* Geriatric nutrition risk index, *CONUT score* The controlling nutritional status score, *PAR* Platelet-albumin ratio, *PNI* Prognostic nutritional index

### Independent risk factors for ESCC

**Fig. 1:**
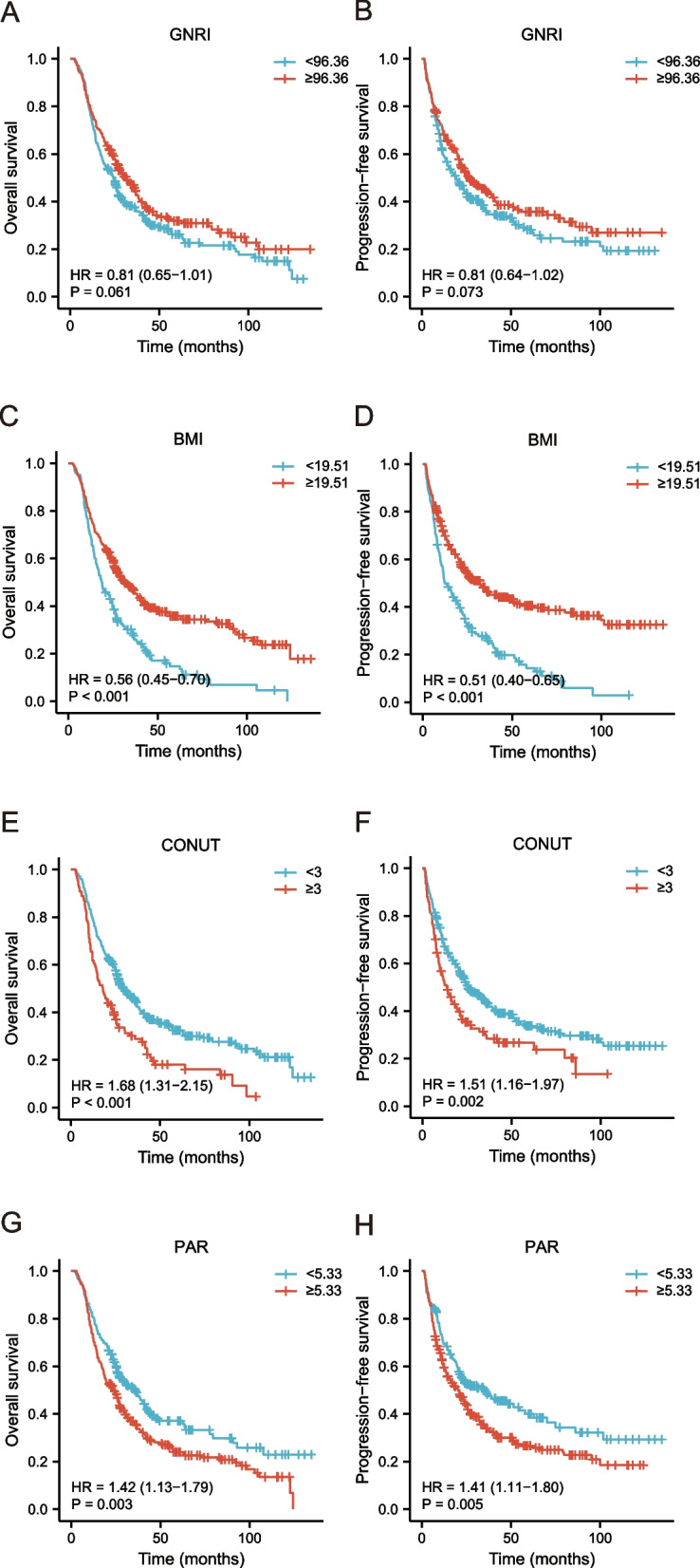
Kaplan–Meier analysis of (**A**, **C**, **E**, **G**) OS and (**B**, **D**, **F**, **H**) PFS were grouped by pre-therapeutic nutritional-related prognostic indicators. OS, overall survival; PFS, progression-free survival; GNRI, geriatric nutrition risk index; BMI, body mass index; CONUT score, the controlling nutritional status score; PAR, platelet-albumin ratio

During the median follow-up period of 24.7 months, 317 elderly ESCC patients died. Univariate analyses revealed that age, tumor length, chemotherapy, N stage, TNM stage, BMI, GNRI, CONUT, PAR, and PNI were potential risk factors for OS and PFS. Multivariate analyses indicated that age, tumor length, N stage, BMI, GNRI, CONUT, and PAR could independently predict prognosis (OS and PFS) in elderly patients with ESCC. Tables 2 and 3 show the specific results of these prognostic analyses. The Kaplan–Meier curves indicated that malnourished elderly individuals had a worse clinical outcome than those without malnutrition (Fig. 1). Figure S1 depicts additional independent prognostic variables affecting OS and PFS.

**Table 2:** Cox regression of fourteen clinical variables with OS

Variables	Univariate analysis		Multivariate analysis	
	HR (95% CI)	*P*-value	HR (95% CI)	*P*-value
Sex				
Male vs. female	1.158(0.913, 1.470)	0.227		
Age (years)				
≥ 76 vs. < 76	1.374(1.100, 1.715)	0.005	1.399(1.094, 1.788)	0.007
Tumor location				
Cervical/upper vs. middle/lower	1.153(0.901, 1.476)	0.257		
Tumor length (cm)				
≥ 5.9 vs. < 5.9	1.531(1.226, 1.912)	< 0.001	1.385(1.095, 1.753)	0.007
RT dose (Gy)				
< 59.96 vs. ≥ 59.96	0.879(0.679, 1.136)	0.323		
Chemotherapy				
Yes vs. no	0.774(0.616, 0.973)	0.028	0.913(0.706, 1.181)	0.488
T stage				
T4 vs. T2/3	0.951(0.759, 1.191)	0.661		
N stage				
N2/3 vs. N0/1	1.555(1.212, 1.995)	0.001	1.464(1.120, 1.913)	0.005
Tumor stage				
Stage III/IVA vs. stage II	1.307(1.002, 1.705)	0.048	1.050(0.785, 1.404)	0.742
BMI				
≥ 19.51 vs. < 19.51	0.560(0.446, 0.704)	< 0.001	0.615(0.485, 0.780)	< 0.001
GNRI				
≥ 96.36 vs. < 96.36	0.809(0.648, 1.010)	0.061	0.772(0.616, 0.967)	0.024
CONUT				
≥ 3 vs. < 3	1.679(1.308, 2.154)	< 0.001	1.488(1.103, 2.008)	0.009
PAR				
≥ 5.33 vs. < 5.33	1.421(1.128, 1.789)	0.003	1.289(1.012, 1.644)	0.040
PNI				
≥ 46.55 vs. < 46.55	0.642(0.515, 0.800)	< 0.001	0.909(0.696, 1.198)	0.486

*OS* Overall survival, *HR* Hazard ratio, *95% CI* 95% confidence interval, *RT* Radiotherapy, *BMI* Body mass index, *GNRI* Geriatric nutrition risk index, *CONUT* Score, the controlling nutritional status score, *PAR* Platelet-albumin ratio, *PNI* Prognostic nutritional index

**Table 3:** Cox regression of fourteen clinical variables with PFS

Variables	Univariate analysis		Multivariate analysis	
	HR (95% CI)	*P*-value	HR (95% CI)	*P*-value
Sex				
Male vs. female	1.100(0.971, 1.247)	0.133		
Age (years)				
≥ 76 vs. < 76	1.302(1.032, 1.642)	0.026	1.344(1.042, 1.733)	0.023
Tumor location				
Cervical/upper vs. middle/lower	1.146(0.885, 1.484)	0.302		
Tumor length (cm)				
≥ 5.9 vs. < 5.9	1.493(1.184, 1.882)	0.001	1.325(1.038, 1.692)	0.024
RT dose (Gy)				
< 59.96 vs. ≥ 59.96	1.086(0.824, 1.433)	0.558		
Chemotherapy				
Yes vs. no	0.809(0.637, 1.028)	0.082	0.941(0.720, 1.231)	0.657
T stage				
T4 vs. T2/3	1.020(0.808, 1.288)	0.866		
N stage				
N2/3 vs. N0/1	1.494(1.153, 1.936)	0.002	1.337(1.013, 1.764)	0.040
Tumor stage				
Stage III/IVA vs. stage II	1.415(1.067, 1.877)	0.016	1.153(0.848, 1.566)	0.364
BMI				
≥ 19.51 vs. < 19.51	0.510(0.403, 0.646)	< 0.001	0.548(0.428, 0.700)	< 0.001
GNRI				
≥ 96.36 vs. < 96.36	0.810(0.643, 1.020)	0.073	0.768(0.608, 0.971)	0.027
CONUT				
≥ 3 vs. < 3	1.513(1.164, 1.968)	0.002	1.428(1.041, 1.958)	0.027
PAR				
≥ 5.33 vs. < 5.33	1.413(1.111, 1.798)	0.005	1.323(1.027, 1.705)	0.031
PNI				
≥ 46.55 vs. < 46.55	0.720(0.572, 0.907)	0.005	1.010(0.764, 1.336)	0.945

*PFS* Progression-free survival, *HR* Hazard ratio, *95% CI* 95% confidence interval, *RT* Radiotherapy, *BMI* Body mass index, *GNRI* Geriatric nutrition risk index, *CONUT* Score, the controlling nutritional status score, *PAR* Platelet-albumin ratio, *PNI* Prognostic nutritional index

In addition, independent prognostic factors were incorporated into the nomogram based on multivariate analysis results. The 1-, 3-, and 5-year OS and PFS calibration curves demonstrated a favourable correlation between observed and predicted values (Figure S2 and Figure S3).

### Pre-therapeutic nutritional prognostic score and new nutritional prognostic index

**Fig. 2:**
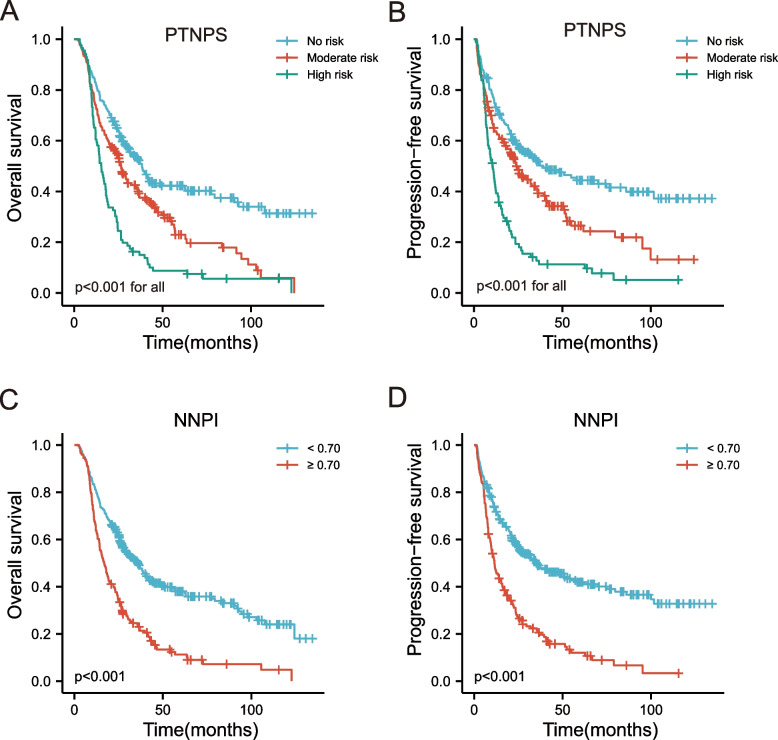
The PTNPS and NNPI for OS and PFS were risk stratified by risk group. **A**, **B** Risk stratification for PTNPS on OS and PFS (*p* < 0.001); **C**, **D** Risk stratification for NNPI on OS and PFS. PTNPS, pre-therapeutic nutritional prognostic score; NNPI, novel nutritional prognostic index; OS, overall survival; PFS, progression-free survival

In this study, the independent nutritional prognostic factors were GNRI, BMI, CONUT, and PAR. The following conditions were considered malnutritional: GNRI < 96.36, BMI < 19.51, CONUT ≥ 3, and PAR ≥ 5.33. The pre-therapeutic nutritional prognostic score (PTNPS) was calculated by adding one point for each of these four risk factors for patients with malnutrition. The patients were divided into three groups according to PTNPS: the no-risk group: PTNPS = 0–1 point; the moderate-risk group: PTNPS = 2 points; and the high-risk group: PTNPS = 3–4 points. 5-year OS rates were 42.3%, 22.9%, and 8.8%, respectively (*p* < 0.001, Fig. 2A); 5-year PFS rates were 44.4%, 26.5%, and 11.3% (*p* < 0.001, Fig. 2B). In addition, we also developed a new nutritional prognostic index (NNPI). The NNPI was determined by multiplying each nutrition-related prognostic factor by its corresponding β coefficient and then summing the results (zero for absence or one for existence). The formula for NNPI is 0.259*GNRI + 0.486*BMI + 0.397*CONUT + 0.254*PAR. The NNPI was divided into high-risk and low-risk subsets dichotomizing by 0.70. The Kaplan–Meier curves showed that the mortality of elderly ESCC patients in the high-risk group was higher than that in the low-risk group (Figs. 2C-D).

### Comparison of the prognostic ability of nutritional indicators

**Fig. 3:**
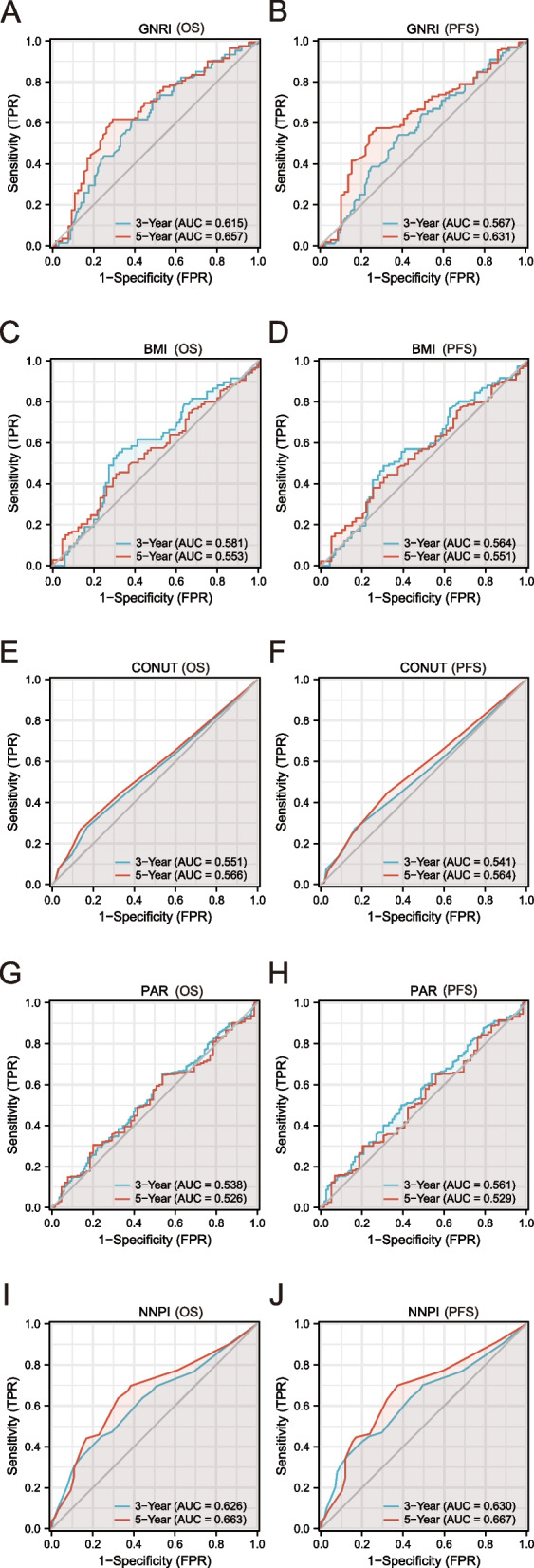
The time-ROC of nutritional-related indicators in older locally advanced ESCC patients. **A**, **B** The time-ROC of GNRI on OS and PFS (3-year and 5-year); **C**, **D** The time-ROC of BMI on OS and PFS (3-year and 5-year); **E**, **F** The time-ROC of CONUT on OS and PFS (3-year and 5-year); **G**, **H** The time-ROC of PAR on OS and PFS (3-year and 5-year); **I**, **J** The time-ROC of NNPI on OS and PFS (3-year and 5-year). time-ROC, the time-dependent receiver operating curve; ESCC, esophageal squamous cell carcinoma; GNRI, geriatric nutrition risk index; OS, overall survival; PFS, progression-free survival; BMI, body mass index; CONUT score, the controlling nutritional status score; PAR, platelet-albumin ratio; NNPI, novel nutritional prognostic index

**Fig. 4:**
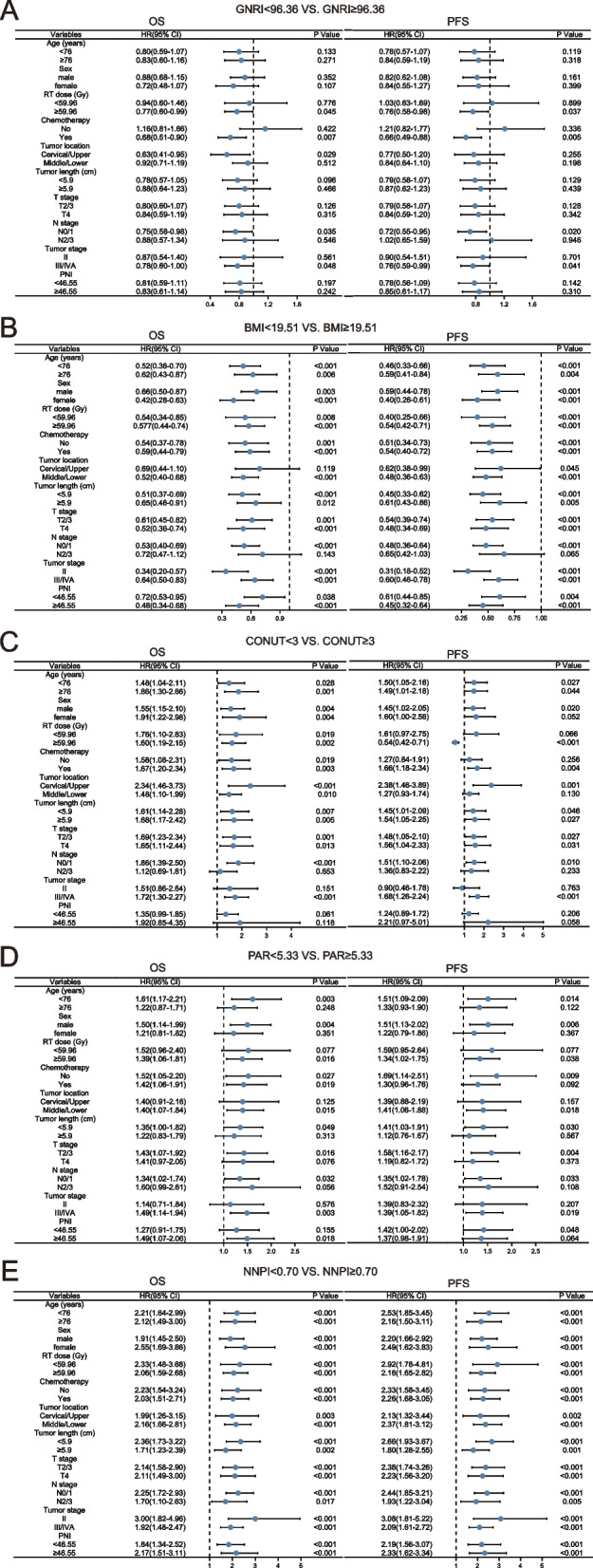
The sub-group analysis of GNRI, BMI, CONUT, PAR, and NNPI in elderly locally advanced ESCC patients. The adjusted factors include age, sex, RT dose, chemotherapy, tumor location, tumor length, T stage, N stage, tumor stage, and PNI. HR, hazard ratio; 95% CI, 95% confidence interval; GNRI, geriatric nutrition risk index; BMI, body mass index; CONUT score, the controlling nutritional status score; PAR, platelet-albumin ratio; ESCC, esophageal squamous cell carcinoma; RT, radiotherapy; PNI, prognostic nutritional index

Comparing the capacity of five nutritional indicators to predict the prognosis of elderly patients with ESCC using C-index and time-ROC. Compared to other nutrition-based indicators, the NNPI had the highest C-index for OS and PFS in older ESCC patients at 3 and 5-years: 0.626 (95% CI, 0.570–0.681) and 0.663 (95% CI, 0.594–0.732), 0.630 (95% CI, 0.573–0.688), and 0.667 (95% CI, 0.594–0.740), respectively (Table 4). Similarly, the NNPI’s area under the ROC curve (AUC) was greater than those of other nutrition-based indicators (Fig. 3). In addition, the forest plot displayed the detailed results of subgroup analyses of GNRI, BMI, GONUT, PAR, and NNPI (Fig. 4). The results suggested that high NNPI was a significant risk factor for increased mortality in elderly patients with ESCC, regardless of age, sex, RT dose, chemotherapy, tumor location, tumor length, T stage, N stage, TNM stage, and PNI. Surprisingly, low GNRI, low BMI, high CONUT, and high PAR were all risk factors for increased mortality in older patients with ≥ 59.96 Gy RT, N stage 0/1, and TNM stage III/IVA. In summary, the NNPI has the best predictive power for prognosis compared with other nutritional indicators and is most applicable to the majority of elderly ESCC patients.

**Table 4:** The C-index of pre-therapeutic nutritional-related prognostic indicators

C-index (95% CI)				
	OS		PFS	
Indicators	3-year	5-year	3-year	5-year
GNRI	0.615(0.532–0.698)	0.657(0.570–0.744)	0.567(0.487–0.682)	0.631(0.544–0.717)
BMI	0.581(0.495–0.668)	0.553(0.462–0.643)	0.564(0.483–0.646)	0.551(0.462–0.640)
CONUT	0.551(0.495–0.607)	0.566(0.496–0.636)	0.541(0.482–0.600)	0.564(0.490–0.639)
PAR	0.538(0.478–0.597)	0.526(0.451–0.601)	0.561(0.498–0.623)	0.529(0.449–0.609)
NNPI	0.626(0.570–0.681)	0.663(0.594–0.732)	0.630(0.573–0.688)	0.667(0.594–0.740)

*95% CI* 95% confidence interval, *GNRI* Geriatric nutrition risk index, *BMI* Body mass index, *CONUT score* The controlling nutritional status score, *PAR* Platelet-albumin ratio, *NNPI* Novel nutritional prognostic index

### Analysis of PTNPS and clinicopathological features in elderly patients with ESCC

**Fig. 5:**
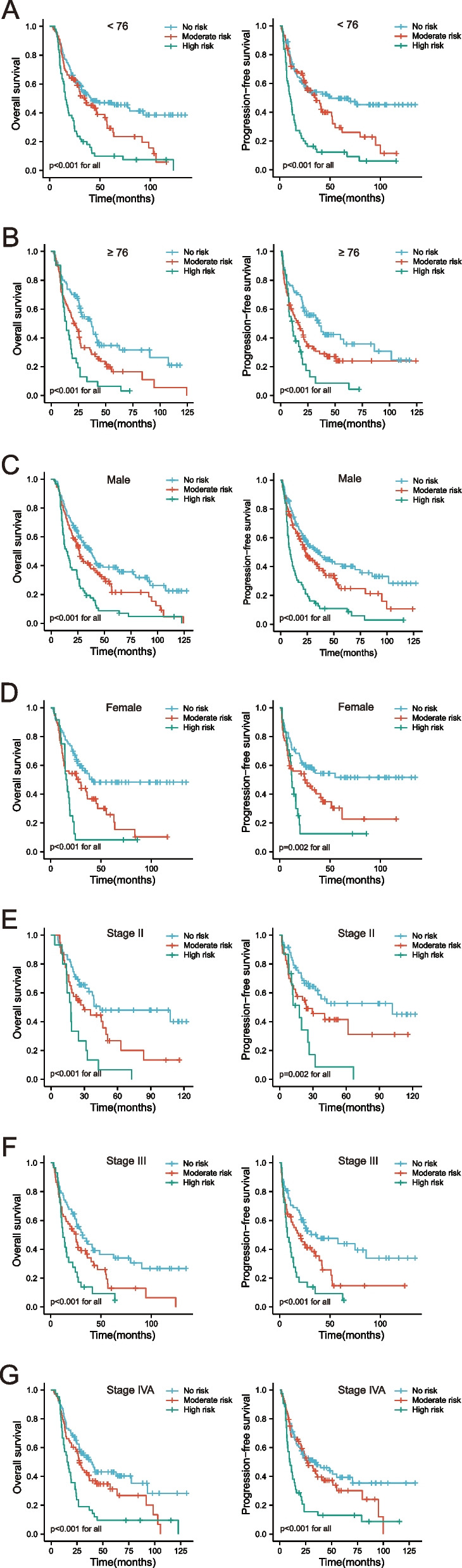
Kaplan–Meier curves are according to age, sex, and tumor stage in different risk groups. **A** OS and PFS of patients with < 76; **B** OS and PFS of patients with ≥ 76; **C** OS and PFS of patients with males; **D** OS and PFS of patients with females; **E** OS and PFS of patients with stage II; **F** OS and PFS of patients with stage III; **G** OS and PFS of patients with stage IVA. OS, overall survival; PFS, progression-free survival

PTNPS classified the patients into three groups: low-risk, moderate-risk, and high-risk. To better understand the impact of different risk groups on prognosis in various clinicopathological characteristics, we performed subgroup analyses of elderly ESCC patients stratified by age, sex, Tumor stage, tumor location, tumor length, RT dose, chemotherapy, and PNI. Figure 5 and S4 showed that elderly ESCC patients in the high-risk group had a poorer OS and PFS than those in the no- or moderate-risk groups.

### Relationship between nutritional indicators and survival

**Fig. 6:**
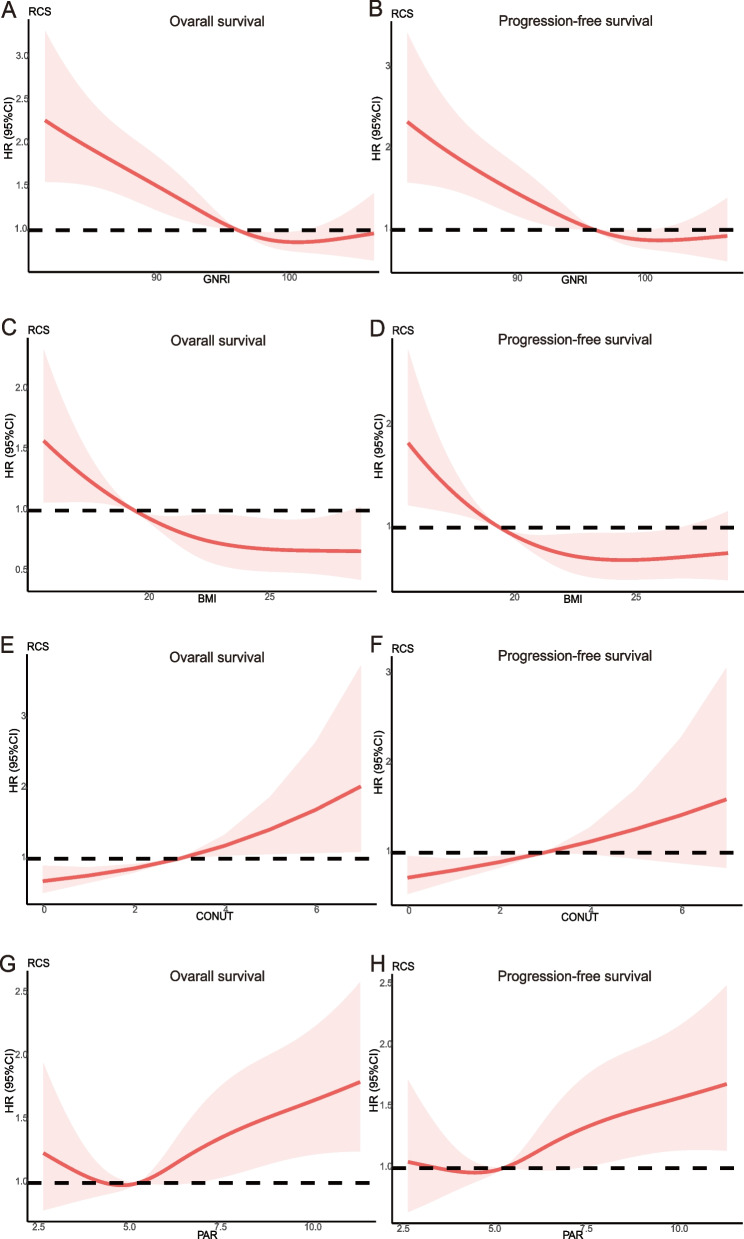
The restricted cubic spline of pre-therapeutic nutritional-related prognostic indicators in elderly locally advanced ESCC patients. **A**, **B** GNRI on OS and PFS; **C**, **D** BMI on OS and PFS; E, F) CONUT on OS and PFS; **G**, **H** PAR on OS and PFS. HR, hazard ratio; GNRI, geriatric nutrition risk index; OS, overall survival; PFS, progression-free survival; BMI, body mass index; CONUT score, the controlling nutritional status score; PAR, platelet-albumin ratio

RCS was performed to categorize the relationship between nutritional indicators and survival. Figure 6A-H presents a linear relationship between nutritional indicators and OS or PFS for elderly ESCC patients, while presenting a non-linear relationship between the BMI and PFS. The GNRI and BMI mortality risks dropped significantly to 96.36 and 19.51, respectively. Patients had higher risks of death when CONUT and PAR increased.

## Discussion

The identification rate of early EC has risen significantly in recent years as a result of improved public health consciousness and medical advancements, but the death rate remains high [19]. Further examination of the causes of mortality indicated that around 25% of cancer-related mortality was attributable to malnutrition rather than the malignancy itself [20, 21]. Literature shows that the prevalence of malnutrition in cancer patients ranges from 30 to 60%, and that it may be much greater in some cancer patients (such as EC and pancreatic cancers) [22–24]. The prognosis for elderly people with inadequate nutritional conditions is frequently dismal. In ESCC patients, it is crucial to comprehend the significance of pre-treatment nutritional indicators and to include these prognostic variables into therapeutic decision-making. Our study found that (i) low GNRI, low BMI, high CONUT, and high PAR were all risk factors for increased mortality in older patients; (ii) the OS and PFS of older patients in the high-risk group were inferior to those in the no- or moderate-risk groups; and (iii) compared to other nutrition-based indicators, the NNPI's C-index and AUC value was the greatest.

Previous studies have demonstrated that GNRI and BMI are useful indicators for predicting the prognosis of older patients with ESCC [25, 26]. The BMI, which is based on weight and height, is an essential diagnostic criterion for cancer-related weight loss [27]. Although multiple studies have reported the impact of preoperative BMI on the prognosis of patients with resected EC, BMI alone is not a valid predictor of survival [28–30]. Traditionally, serum albumin levels have been seen as an indicator of malnutrition and a biomarker of protein stores [31]. However, BMI and serum albumin have limited value as measures of a patient's nutritional status. In numerous types of cancer, the GNRI, which is comprised of a patient's serum albumin level, height, and weight, has been demonstrated to be a more accurate indicator of nutritional status than BMI and serum albumin [24, 25, 31, 32]. Our results showed that GNRI has a higher C-index and time-ROC value than BMI, indicating that it is a more reliable predictor of OS and PFS than BMI. Wang et al. found that GNRI was an independent predictive factor for OS and PFS. Additionally, GNRI can aid in risk stratification for elderly patients receiving dRT or dCRT [33]. Interestingly, low GNRI was a risk factor for increased mortality in older patients with ≥ 59.96 Gy RT, receiving chemotherapy, N stage 0/1, and TNM stage III/IVA in our subgroup analyses. The original purpose of the GNRI was to assess the nutritional condition of elderly patients. Tang et al. found that GNRI is an effective method for predicting the long-term prognosis, providing a theoretical base for early nutritional management in elderly patients with colorectal cancer [34].

Recent studies have demonstrated that the CONUT score, a helpful indicator of immune-nutrition status, is a valid prognostic indicator in many malignancies [35, 36]. Among 373 ESCC patients who underwent radical resection, one study revealed that those with moderate or severe CONUT scores had a poor prognosis [36]. Another study showed that PAR is a potential independent predictor ofprognosis in ESCC patients treated with dCRT [15]. Consistent with earlier studies, our findings revealed that high CONUT and high PAR had worse survival outcomes in elderly patients with ESCC. Nonetheless, the precise mechanism behind the association between CONUT and PAR and tumor prognosis remains unknown. CONUT is known to derive from three hematological indicators (total cholesterol, albumin levels, and lymphocytes), representing calorie expenditure, protein stores, and poor immune defense, respectively. PAR is composed of albumin and platelets, which represent protein storage and systemic inflammatory response, respectively. Cholesterol, an important part of the cell membrane, is involved in numerous biological signaling pathways. Hypocholesterolemia resulting from increased cholesterol absorption by tumor cells impairs the transmission of transmembrane signals [37]. Serum albumin levels are regarded as a reliable predictor of nutritional condition and systemic inflammation. According to studies, hypoproteinemia has a poor prognosis by causing a series of inflammatory cytokines to be released, including IL-6, TNF-α [38]. According to previous research, malnutrition may diminish lymphocyte maturity and the amount of lymphocytes in circulation. Lymphopenia leads to an inadequate immune response, which impacts the prognosis of cancer [39]. High platelet counts can influence the development of malignancies and lead to thrombocytosis, which is a negative factor influencing the clinical outcome of patients [40]. Therefore, combining the above components, CONUT and PAR may provide a better balance of immune, inflammatory, and nutritional status.

Malnutrition in elderly ESCC patients receiving dCRT or dRT may be caused by dysphagia, insufficient nutrient intake due to tumor obstruction, and other factors. Poor nutritional status may be easily aggravated during dCRT or dRT. Studies have shown that malnourished elderly people are susceptible to radiation esophagitis, which further reduces their nutritional intake and thus aggravates their malnutrition [41]. This study demonstrates that a higher nutritional risk is associated with shorter survival in older patients with locally advanced ESCC. It is widely recognized that malnutrition is a negative prognostic factor in a variety of malignancies, since it can impair the immunological system of the patient and promote tumor progression [42]. In addition, malnutrition can influence the patient's treatment effect and reaction, as well as diminish the patient's tolerance and compliance with therapy [43, 44]. Consequently, screening of malnutrition before to therapy facilitates early nutritional intervention and may improve outcomes, particularly for this subpopulation. Moreover, nutritional interventions can decrease the adverse effects of anticancer medications.

Our study has a few limitations. First, this was a retrospective, non-random, single-center study with potential selection bias. A larger prospective multicenter study is warranted to assess the predictive value of pre-therapeutic nutritional indicators in older individuals with ESCC. Second, there are no standardized cut-off values for each indicator, leading to inaccurate screening. It is hoped that in the future generally accepted cut-off values can be identified and these nutritional indicators can be used as tools for early nutritional risk screening. Third, only older ESCC patients treated with dCRT or dRT were included in this study, and the results may not be applicable to the overall situation of older ESCC patients treated with other therapies (such as surgery and immunotherapy). Finally, we cannot rule out other confounding factors beyond examination that may be related to nutritional status or survival. Despite these limitations, this study may serve as a significant reference for predicting survival in older ESCC patients.

## Conclusions

The GNRI, BMI, CONUT score, and PAR can be considered as objective evaluation tools to assess the risk of nutrition-related mortality in elderly patients with ESCC receiving dCRT or dRT. Compared to the four indicators, the NNPI provides the greatest predictive value for prognosis and is most appropriate for the majority of senior ESCC patients. Lastly, we discovered that elderly patients with a greater nutritional risk had a poorer outcome.

## Supplementary Information

**Additional file 1:** **FigureS1. **Kaplan-Meier analysis of age, tumor length, andN stage for (A, C, E) OS and (B, D, F) PFS. OS, overall survival; PFS,progression-free survival; GNRI, geriatric nutrition risk index; BMI, body massindex; CONUT score, the controlling nutritional status score; PAR,platelet-albumin ratio. **FigureS2. **(A) Prediction nomogram for 1-year, 3-year, and 5-year OS. (B-D)Calibration curves depicting the probability of 1-year, 3-year, and 5-year OSbetween the prediction and the actual observation. The X-axis represents theprobability predicted by the nomogram, while the Y-axis represents the actualobservation. **FigureS3. **(A)Prediction nomogram for 1-year, 3-year, and 5-year PFS. (B-D) Calibrationcurves depicting the probability of 1-year, 3-year, and 5-year PFS between theprediction and the actual observation. The X-axis represents the probabilitypredicted by the nomogram, while the Y-axis represents the actual observation. **FigureS4. **Kaplan-Meier curves are according to tumorlocation, tumor length, RT dose, chemotherapy, and PNI in different riskgroups. (A) OS and PFS of patients with tumor locations located in thecervical/upper; (B) OS and PFS of patients with tumor locations located in themiddle/lower; (C) OS and PFS of patients with tumor lengths < 5.9 cm; (D) OSand PFS of patients with tumor lengths ≥ 5.9 cm; (E) OS and PFS of patientswith RT dose < 59.96 Gy; (F) OS and PFS of patients with RT dose ≥ 59.96 Gy;(G) OS and PFS of patients without chemotherapy; (H) OS and PFS of patientswith chemotherapy; (I) OS and PFS of patients with PNI < 46.55; (J) OS andPFS of patients with PNI ≥ 46.55. RT, radiotherapy; PNI, prognostic nutritionalindex; OS, overall survival; PFS, progression-free survival.

## Data Availability

The data supporting the results of this study can be provided by the corresponding author upon reasonable request.

## Abbreviations

EC: Esophageal cancer
ESCC: Esophageal squamous cell carcinoma
GNRI: Geriatric nutrition risk index
BMI: Body mass index
CONUT score: The controlling nutritional status score
PAR: Platelet-albumin ratio
PNI: Prognostic nutritional index
dCRT: Definitive chemoradiotherapy
dRT: Definitive radiotherapy
PTNPS: Pre-therapeutic nutritional prognostic score
NNPI: Novel nutritional prognostic index
IMRT: Intensity-modulated radiotherapy
3D-CRT: 3-Dimensional conventional radiotherapy
OAR: Organs at risk
CT: Computed tomography
FDG-PET: 18F-fluorodeoxyglucose positron emission tomography
CW: Current weight
IBW: Ideal body weight
OS: Overall survival
PFS: Progression-free survival
ROC: The receiver operating curve
time-ROC: The time-dependent receiver operating curve
RCS: Restricted cubic splines
AUC: Area under the ROC curve

## References

[1] Sung H (2021). Global Cancer Statistics 2020: GLOBOCAN Estimates of Incidence and Mortality Worldwide for 36 Cancers in 185 Countries. CA Cancer J Clin.

[2] Pennathur A, Gibson MK, Jobe BA, Luketich JD (2013). Oesophageal carcinoma. Lancet.

[3] Faiz Z (2012). Increased resection rates and survival among patients aged 75 years and older with esophageal cancer: a Dutch nationwide population-based study. World J Surg.

[4] van Blankenstein M, Looman CW, Siersema PD, Kuipers EJ, Coebergh JW (2007). Trends in the incidence of adenocarcinoma of the oesophagus and cardia in the Netherlands 1989–2003. Br J Cancer.

[5] Anandavadivelan P, Lagergren P (2016). Cachexia in patients with oesophageal cancer. Nat Rev Clin Oncol.

[6] Barker LA, Gout BS, Crowe TC (2011). Hospital malnutrition: prevalence, identification and impact on patients and the healthcare system. Int J Environ Res Public Health.

[7] McMillan DC (2009). Systemic inflammation, nutritional status and survival in patients with cancer. Curr Opin Clin Nutr Metab Care.

[8] Rock CL (2012). Nutrition and physical activity guidelines for cancer survivors. CA Cancer J Clin.

[9] Liu X (2015). Prognostic significance of pretreatment serum levels of albumin, LDH and total bilirubin in patients with non-metastatic breast cancer. Carcinogenesis.

[10] Mayne ST, Playdon MC, Rock CL (2016). Diet, nutrition, and cancer: past, present and future. Nat Rev Clin Oncol.

[11] Lv GY, An L, Sun DW (2019). Geriatric nutritional risk index predicts adverse outcomes in human malignancy: a meta-analysis. Dis Markers.

[12] Toyokawa T (2016). The pretreatment Controlling Nutritional Status (CONUT) score is an independent prognostic factor in patients with resectable thoracic esophageal squamous cell carcinoma: results from a retrospective study. BMC Cancer.

[13] Feng J, Wang L, Yang X, Chen Q, Cheng X (2022). The usefulness of pretreatment controlling nutritional status score for predicting recurrence in patients with esophageal squamous cell carcinoma undergoing neoadjuvant immunochemotherapy: A real-world study. Front Immunol.

[14] Okadome K (2020). Prognostic Nutritional Index, Tumor-infiltrating Lymphocytes, and Prognosis in Patients with Esophageal Cancer. Ann Surg.

[15] Zheng, Z., Zhu, H. & Cai, H. Preoperative Prognostic Nutritional Index Predict Survival in Patients With Resectable Esophageal Squamous Cell Carcinoma. Front Nutr (2022)9:82483910.3389/fnut.2022.824839. 10.3389/fnut.2022.824839 PMC9043690 35495910

[16] Huang Z (2022). Prognostic significance of platelet-to-albumin ratio in patients with esophageal squamous cell carcinoma receiving definitive radiotherapy. Sci Rep.

[17] Dogan Akagunduz, D. & Turker, P. F. Nutritional Support in Older Patients with Esophageal Cancer Undergoing Chemoradiotherapy. Nutr Cancer (2022) 74:3634–3639, doi:10.1080/01635581.2022.2096245. 10.1080/01635581.2022.2096245 35786221

[18] Short MW, Burgers KG, Fry VT (2017). Esophageal Cancer. Am Fam Physician.

[19] Smyth EC (2017). Oesophageal cancer Nat Rev Dis Primers.

[20] Caccialanza R (2020). Unmet needs in clinical nutrition in oncology: a multinational analysis of real-world evidence. Ther Adv Med Oncol.

[21] Senesse P (2008). Nutritional support during oncologic treatment of patients with gastrointestinal cancer: who could benefit?. Cancer Treat Rev.

[22] Hebuterne X (2014). Prevalence of malnutrition and current use of nutrition support in patients with cancer. JPEN J Parenter Enteral Nutr.

[23] Tobert CM, Mott SL, Nepple KG (2018). Malnutrition Diagnosis during Adult Inpatient Hospitalizations: Analysis of a Multi-Institutional Collaborative Database of Academic Medical Centers. J Acad Nutr Diet.

[24] Pirlich M (2006). The German hospital malnutrition study. Clin Nutr.

[25] Bo, Y. et al*.* The Geriatric Nutritional Risk Index Predicts Survival in Elderly Esophageal Squamous Cell Carcinoma Patients with Radiotherapy. PLoS On*e *(2016) 11:e0155903:10.1371/journal.pone.0155903. 10.1371/journal.pone.0155903 PMC4873221 27196126

[26] Gu, Y. M. *et al.* The prognostic impact of preoperative body mass index changes for patients with esophageal squamous cell carcinoma who underwent esophagectomy: A large-scale long-term follow-up cohort study. Front Nutr (2022);9:947008. 10.3389/fnut.2022.947008. 10.3389/fnut.2022.947008 PMC9678912 36424925

[27] Martin L (2015). Diagnostic criteria for the classification of cancer-associated weight loss. J Clin Oncol.

[28] Zhang SS (2013). The impact of body mass index on complication and survival in resected oesophageal cancer: a clinical-based cohort and meta-analysis. Br J Cancer.

[29] Deng, H. Y. *et al.* High BMI has no impact on the survival of Chinese patients with lower thoracic esophageal adenocarcinoma treated with curative esophagectomy: a propensity score-matched study. Dis Esophagus (2019);32. 10.1093/dote/doy059. 10.1093/dote/doy059 29931316

[30] Hasegawa T (2015). Impact of body mass index on surgical outcomes after esophagectomy for patients with esophageal squamous cell carcinoma. J Gastrointest Surg.

[31] Gupta R, Ihmaidat H (2003). Nutritional effects of oesophageal, gastric and pancreatic carcinoma. Eur J Surg Oncol.

[32] Takahashi, M. *et al.* Comparison of three nutritional scoring systems for outcomes after complete resection of non-small cell lung cancer. J Thorac Cardiovasc Surg (2021);162, 1257–1268 e1253. 10.1016/j.jtcvs.2020.06.030. 10.1016/j.jtcvs.2020.06.030 32771232

[33] Oh J (2020). Association between nutritional risk index and outcomes for head and neck cancer patients receiving concurrent chemo-radiotherapy. Head Neck.

[34] Tang S (2020). The Value of Geriatric Nutritional Risk Index in Evaluating Postoperative Complication Risk and Long-Term Prognosis in Elderly Colorectal Cancer Patients. Cancer Manag Res.

[35] Li Y (2020). Prognostic significance of the controlling nutritional status (CONUT) score in epithelial ovarian cancer. Int J Gynecol Cancer.

[36] Yoshida N (2017). Preoperative controlling nutritional status (CONUT) is useful to estimate the prognosis after esophagectomy for esophageal cancer. Langenbecks Arch Surg.

[37] Zhou P, Li B, Liu B, Chen T, Xiao J (2018). Prognostic role of serum total cholesterol and high-density lipoprotein cholesterol in cancer survivors: A systematic review and meta-analysis. Clin Chim Acta.

[38] Goh SL, De Silva RP, Dhital K, Gett RM (2015). Is low serum albumin associated with postoperative complications in patients undergoing oesophagectomy for oesophageal malignancies?. Interact Cardiovasc Thorac Surg.

[39] Damen PJJ (2021). The influence of severe radiation-induced lymphopenia on overall survival in solid tumors: a systematic review and meta-analysis. Int J Radiat Oncol Biol Phys.

[40] Horsted, F., West, J. & Grainge, M. J. Risk of venous thromboembolism in patients with cancer: a systematic review and meta-analysis. PLoS Med (2012);9:e1001275. 10.1371/journal.pmed.1001275. 10.1371/journal.pmed.1001275 PMC3409130 22859911

[41] Dong J (2020). Baseline nutritional status could be a predictor for radiation esophagitis in esophageal cancer patients undergoing radiotherapy. Ann Transl Med.

[42] Yang Y (2018). Platelet to lymphocyte ratio is a predictive marker of prognosis and therapeutic effect of postoperative chemotherapy in non-metastatic esophageal squamous cell carcinoma. Clin Chim Acta.

[43] Grivennikov SI, Greten FR, Karin M (2010). Immunity, inflammation, and cancer. Cell.

[44] Lobo DN (2020). Perioperative nutrition: Recommendations from the ESPEN expert group. Clin Nutr.

