# Supplementary material for: Prognostic value of pre-therapeutic nutritional risk factors in elderly patients with locally advanced esophageal squamous cell carcinoma receiving definitive chemoradiotherapy or radiotherapy
Source: BMC Cancer. 2023 Jun 28;23:597. doi: 10.1186/s12885-023-11044-5 (PMC10303855; doi:10.1186/s12885-023-11044-5)
Supplement: Supplementary file 1 — Additional file 1: FigureS1. Kaplan-Meier analysis of age, tumor length, andN stage for (A, C, E) OS and (B, D, F) PFS. OS, overall survival; PFS,progression-free survival; GNRI, geriatric nutrition risk index; BMI, body massindex; CONUT score, the controlling nutritional status score; PAR,platelet-albumin ratio. FigureS2. (A) Prediction nomogram for 1-year, 3-year, and 5-year OS. (B-D)Calibration curves depicting the probability of 1-year, 3-year, and 5-year OSbetween the prediction and the actual observation. The X-axis represents theprobability predicted by the nomogram, while the Y-axis represents the actualobservation. FigureS3. (A)Prediction nomogram for 1-year, 3-year, and 5-year PFS. (B-D) Calibrationcurves depicting the probability of 1-year, 3-year, and 5-year PFS between theprediction and the actual observation. The X-axis represents the probabilitypredicted by the nomogram, while the Y-axis represents the actual observation. FigureS4. Kaplan-Meier curves are according to tumorlocation, tumor length, RT dose, chemotherapy, and PNI in different riskgroups. (A) OS and PFS of patients with tumor locations located in thecervical/upper; (B) OS and PFS of patients with tumor locations located in themiddle/lower; (C) OS and PFS of patients with tumor lengths < 5.9 cm; (D) OSand PFS of patients with tumor lengths ≥ 5.9 cm; (E) OS and PFS of patientswith RT dose < 59.96 Gy; (F) OS and PFS of patients with RT dose ≥ 59.96 Gy;(G) OS and PFS of patients without chemotherapy; (H) OS and PFS of patientswith chemotherapy; (I) OS and PFS of patients with PNI < 46.55; (J) OS andPFS of patients with PNI ≥ 46.55. RT, radiotherapy; PNI, prognostic nutritionalindex; OS, overall survival; PFS, progression-free survival. [file 12885_2023_11044_MOESM1_ESM.docx]

**
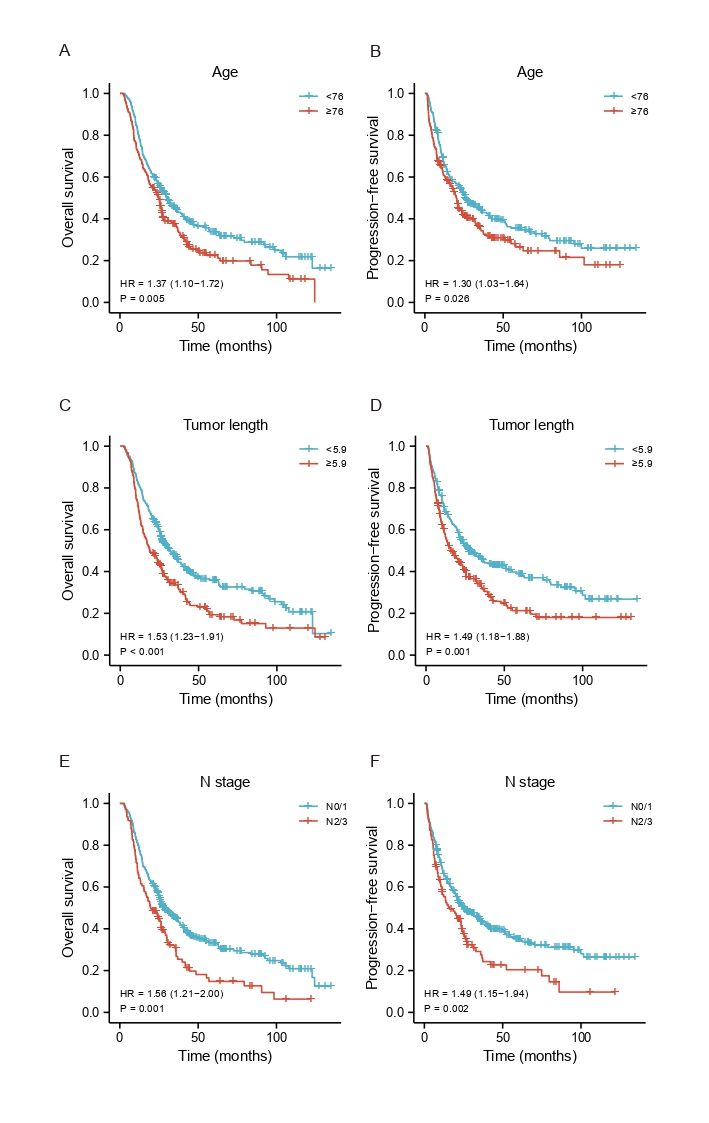
**

**Figure S1** Kaplan-Meier analysis of age, tumor length, and N stage for (A, C, E) OS and (B, D, F) PFS. OS, overall survival; PFS, progression-free survival; GNRI, geriatric nutrition risk index; BMI, body mass index; CONUT score, the controlling nutritional status score; PAR, platelet-albumin ratio.


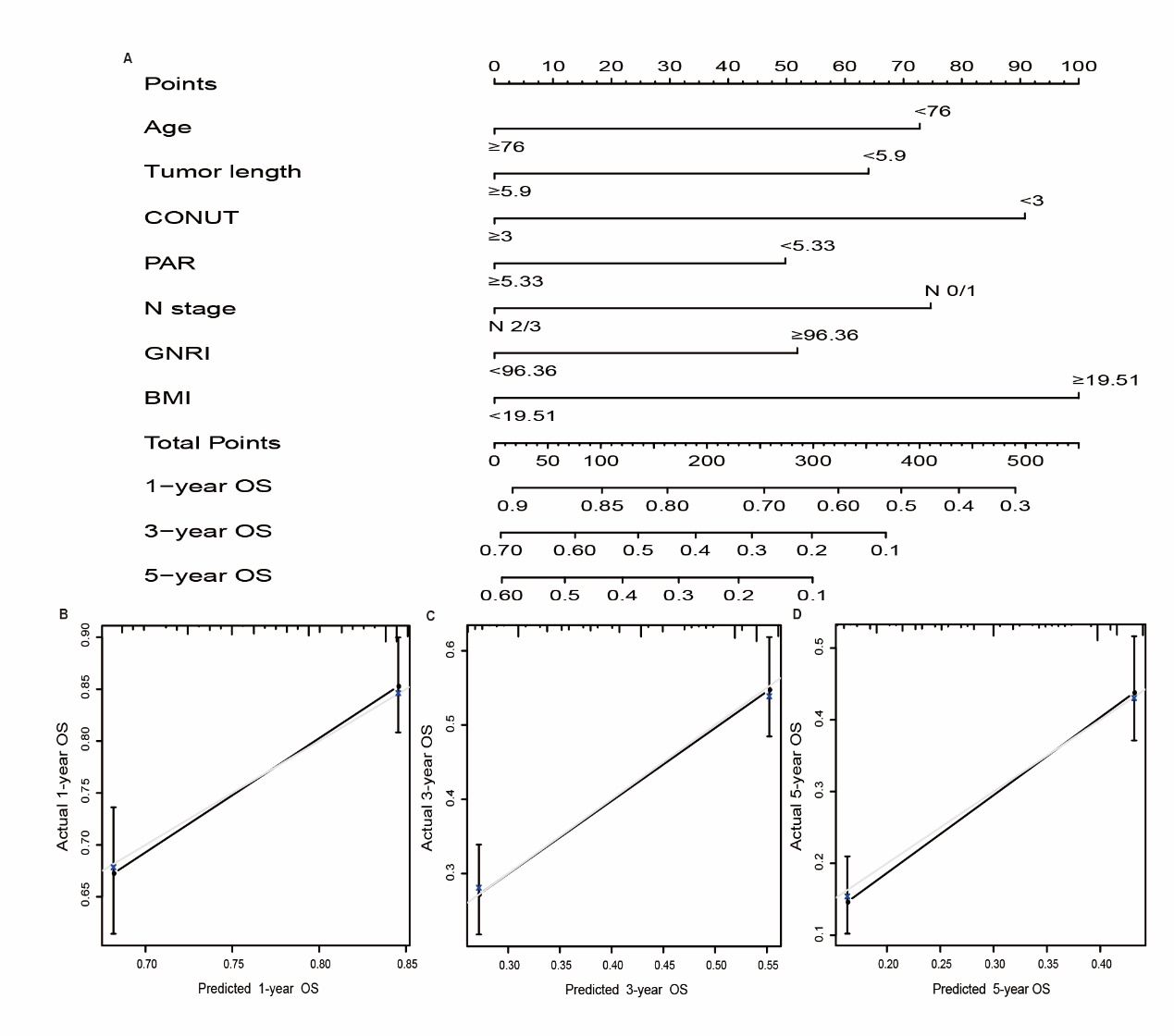


**Figure S2** (A) Prediction nomogram for 1-year, 3-year, and 5-year OS. (B-D) Calibration curves depicting the probability of 1-year, 3-year, and 5-year OS between the prediction and the actual observation. The X-axis represents the probability predicted by the nomogram, while the Y-axis represents the actual observation.


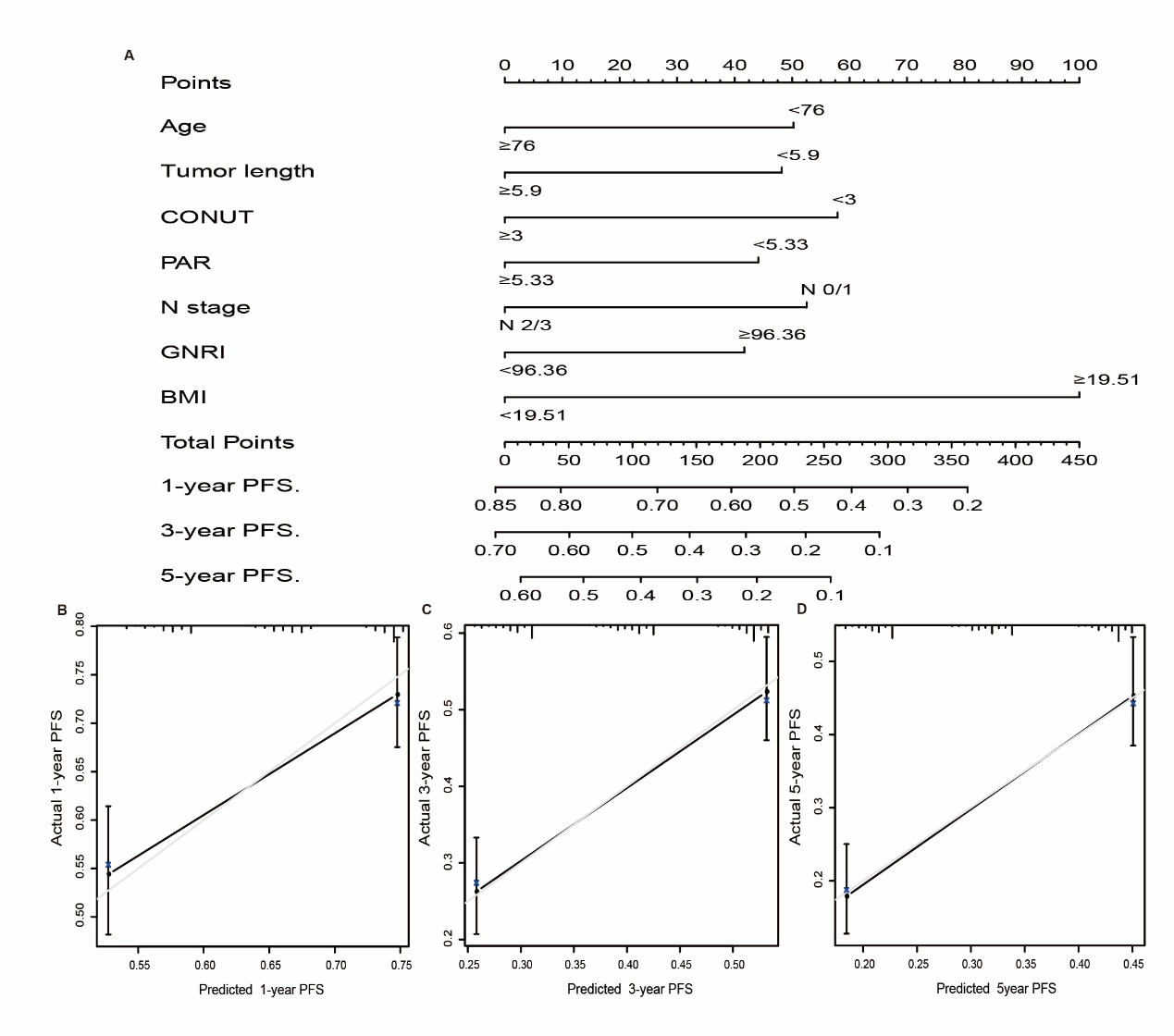


**Figure S3** (A) Prediction nomogram for 1-year, 3-year, and 5-year PFS. (B-D) Calibration curves depicting the probability of 1-year, 3-year, and 5-year PFS between the prediction and the actual observation. The X-axis represents the probability predicted by the nomogram, while the Y-axis represents the actual observation.

**
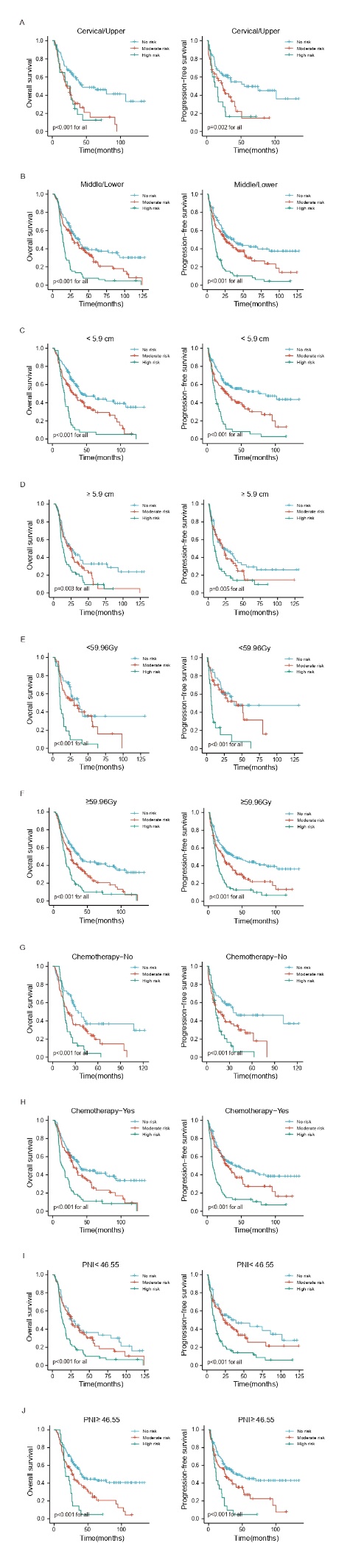
**

**Figure S4** Kaplan-Meier curves are according to tumor location, tumor length, RT dose, chemotherapy, and PNI in different risk groups. (A) OS and PFS of patients with tumor locations located in the cervical/upper; (B) OS and PFS of patients with tumor locations located in the middle/lower; (C) OS and PFS of patients with tumor lengths < 5.9 cm; (D) OS and PFS of patients with tumor lengths ≥ 5.9 cm; (E) OS and PFS of patients with RT dose < 59.96 Gy; (F) OS and PFS of patients with RT dose ≥ 59.96 Gy; (G) OS and PFS of patients without chemotherapy; (H) OS and PFS of patients with chemotherapy; (I) OS and PFS of patients with PNI < 46.55; (J) OS and PFS of patients with PNI ≥ 46.55. RT, radiotherapy; PNI, prognostic nutritional index; OS, overall survival; PFS, progression-free survival.
